# The global academic distribution and changes in research hotspots of artificial intelligence in inflammatory bowel disease since 2000

**DOI:** 10.3389/fmed.2025.1600291

**Published:** 2025-07-11

**Authors:** Likang Xu, Jinzhao Zou, Chao Sun, Gong Chen, Sujun Gao

**Affiliations:** ^1^Department of Clinical Medicine, Medical College, Yangzhou University, Yangzhou, China; ^2^Department of Medical Imaging, Northern Jiangsu People’s Hospital, Clinical Medical College of Yangzhou University, Yangzhou, China; ^3^Digestive Department of Northern Jiangsu People’s Hospital, Clinical Medical College of Yangzhou University, Yangzhou, China

**Keywords:** artificial intelligence, inflammatory bowel disease, Crohn’s disease, ulcerative colitis, bibliometric, Web of Science

## Abstract

**Background:**

Artificial intelligence (AI) has gained widespread attention because of its potential applications in the clinical management of inflammatory bowel disease (IBD). However, bibliometric analyses of the literature published in this field are currently lacking. This study aimed to characterize the development trends and identify research hotspots in the application of AI to IBD through a bibliometric approach.

**Methods:**

Literature related to the application of AI in IBD, published between 2000 and 2024, was retrieved from the Web of Science Core Collection. Microsoft Office Excel 2021 was used to analyze and visualize the annual number of publications. Charticulator was utilized to create country cross chord charts. CiteSpace was employed to visualize collaboration networks among authors, institutions, and countries, generate timeline visualizations and perform a burst analysis of references and keywords.

**Results:**

The bibliometric analysis included 1,136 publications published between the years 2000 and 2024. The number of annual publications showed a rapid growth trend. The USA (*n* = 337) and Harvard University (*n* = 47) had the most published papers. Stidham, Ryan (*n* = 21) published the highest number of articles. The core journals shaping the field included Scientific Reports, Inflammatory Bowel Diseases, and PLoS One. The timeline view and burst analysis of references and keywords revealed that the research hotspots focused on radiomics, endoscopy, natural language processing (NLP), and personalized medicine after 2020.

**Conclusion:**

A growing number of researchers have engaged in exploring the application of AI in IBD, with the USA contributing the most to this field. Currently, the research hotspots mainly involve radiomics, endoscopy, NLP, and personalized medicine. This study provides valuable information for researchers evaluating the application of AI in IBD.

## 1 Introduction

Inflammatory bowel diseases (IBD), including Crohn’s disease (CD) and ulcerative colitis (UC), are characterized by chronic intestinal inflammation (1). The clinical symptoms of IBD include abdominal pain, diarrhea, weight loss, and bloody stools. The disease can lead to serious complications such as stricture, fistula, infection, and even cancer (2). Moreover, the incidence of IBD is increasing globally each year, particularly in developing countries. Consequently, IBD has become a major global public health concern (3, 4). In recent years, immunomodulators and biologic agents have led to significant advancements in the treatment of IBD. However, due to the high clinical heterogeneity of the disease and individual variations in patient response to treatment, challenges persist (5). This highlights the urgent need to develop more precise diagnostic, assessment, and treatment strategies.

Artificial intelligence (AI) has shown great potential in the diagnosis and treatment of IBD. AI can effectively identify the early signs of disease, predict disease progression, and assist in the development of personalized treatment plans (6–8). In addition, AI has demonstrated significant potential for drug discovery and facilitated the development of new therapies (9). Moreover, the continuous expansion of datasets and optimization of algorithms will further advance the application of AI in developing personalized therapies for IBD.

Several review articles have explored the application of AI in IBD; however, these reviews often lack objective quantitative analyses and rely overly on the subjective understanding of researchers. Consequently, these reviews are often variable and subjective when presenting research findings. In contrast, bibliometrics, as a quantitative analysis tool, can objectively and systematically analyze authors, institutions, countries, references, and keywords within a field (10). Hence, bibliometrics have become an essential tool for tracking research trends and predicting future directions and is widely applied in medical research. However, a comprehensive analysis of the literature in this field is lacking.

Therefore, this study aimed to utilize bibliometric methods to systematically analyze research on AI in IBD since the 21st century, identify major research contributors, examine the distribution of current research outcomes, identify research hotspots, and explore the frontiers of this field, thereby establishing a systematic and comprehensive knowledge framework.

## 2 Materials and methods

### 2.1 Data sources and retrieval strategies

The Web of Science Core Collection (WoSCC) is globally recognized as one of the most authoritative databases and is widely considered the preferred platform for bibliometric analysis (11). The data analyzed in this study included all literature on the application of AI in IBD since 2000, which was retrieved on January 11, 2025. The search strategy is described in Supplementary material 1.

**Figure 1:**
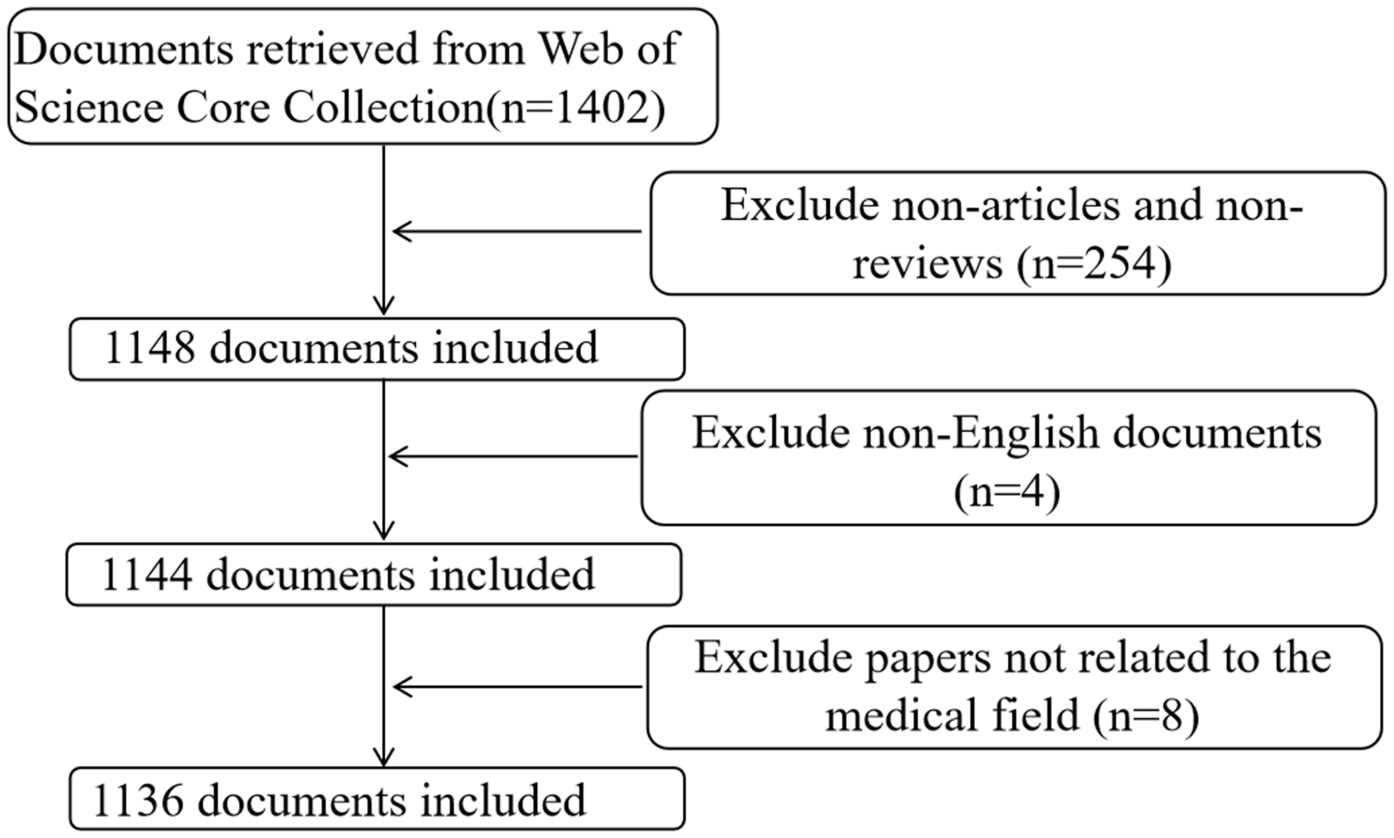
Flowchart of the search stage in the study.

The initial search results retrieved 1,402 documents. After filtering the data to include only “article” and “review article” types, 1,148 documents were obtained. After excluding four non-English documents and eight documents from the fields of zoology and veterinary science, 1,136 documents were included in the final analysis. The search and screening process is illustrated in Figure 1. The data were exported in “plain text” format from the “Full Record and Cited References” section of the WoSCC platform. In addition, citation reports for the top 10 most-cited articles; the top 10 most prolific authors, institutions, and countries; and the top 15 journals by publication volume were obtained from the WoSCC.

### 2.2 Data processing and analysis

Data deduplication was performed using CiteSpace software (6.3. R3, Advanced), and no duplicate records were obtained. Keywords with similar meanings, singular and plural forms, and abbreviations were merged. For example, “inflammatory bowel disease (IBD),” “IBD,” “inflammatory bowel diseases,” and “inflammatory bowel disease” were merged into “inflammatory bowel disease.” CiteSpace was used to visualize collaboration networks among authors, institutions, and countries, generate keyword and reference timeline visualization maps, and identify keywords and references with the strongest citation bursts. Microsoft Office Excel 2021 and Charticulator were used to visualize the annual number of publications and construct country cross chord charts, respectively.

### 2.3 Interpretation of charts and indicators

Each node in the collaboration network, using by CiteSpace, represents an entity. The color of a node indicates the year of its first appearance. The lines between nodes indicate the degree of collaboration between them (12). A notable advantage of network visualization is its ability to quickly reveal the structure and relationships among the nodes and accurately highlight key and influential nodes (13). In the burst analysis, the timeline from 2002 to 2024 is represented by lines where light-green segments indicate periods when the entity has not yet appeared in the dataset, dark-green segments denote active periods of research, and red segments indicate burst periods of research (14).

The average number of citations per publication (AC/P) can be used to measure the citation impact of a scholar, institution, or country within a specific period, thereby assessing its research influence (15). The *H*-index is used to evaluate the academic achievements of individual researchers and has been increasingly applied to measure the research impact of institutions, countries, and journals (16). The modularity (*Q* value) and silhouette (*S* value) values were used to evaluate the community structure and clustering quality of the network (17). A *Q* value >0.3 indicates a significant clustering structure. The *S* value is typically used to assess the quality and cohesion of the network clustering. An *S* value >0.7 indicates good clustering quality.

## 3 Results

### 3.1 Scientific output

**Figure 2:**
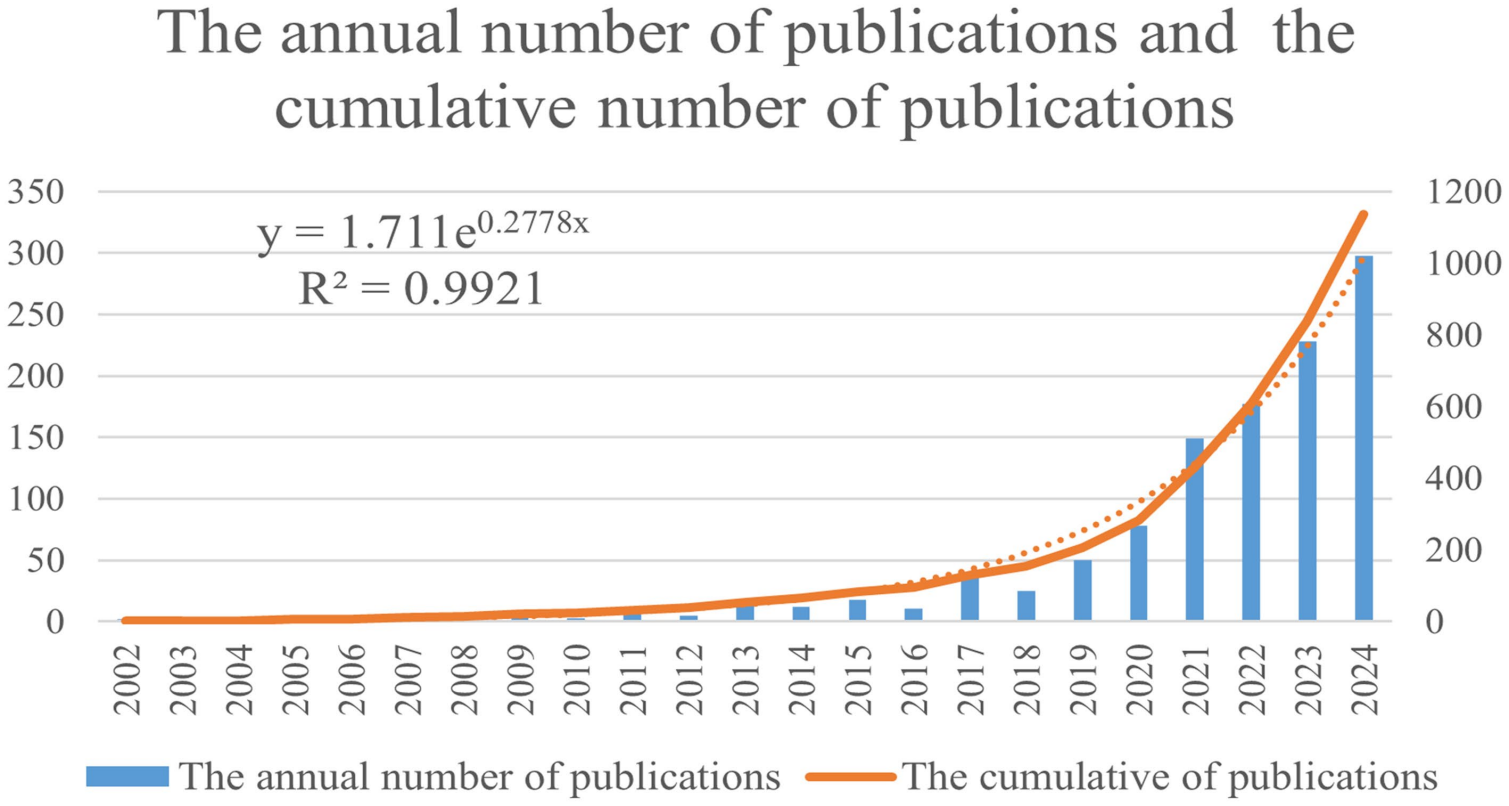
Annual and cumulative output about the application of AI in IBD related publications from 2002 to 2024.

A total of 1,136 publications were included in the analysis, comprising 937 articles and 199 reviews. Figure 2 illustrates the trend in the annual number of publications and cumulative number of publications. The results indicate that research on the application of AI in IBD is growing rapidly. In 2013, the annual number of publications exceeded 10 for the first time, which surpassed 100 by 2021. In 2024, the annual number of publications reached 427. The exponential regression analysis (*R*^2^ = 0.9921) indicated that the cumulative number of publications showed a continuous exponential growth trend.

### 3.2 Analysis of authors

**Figure 3:**
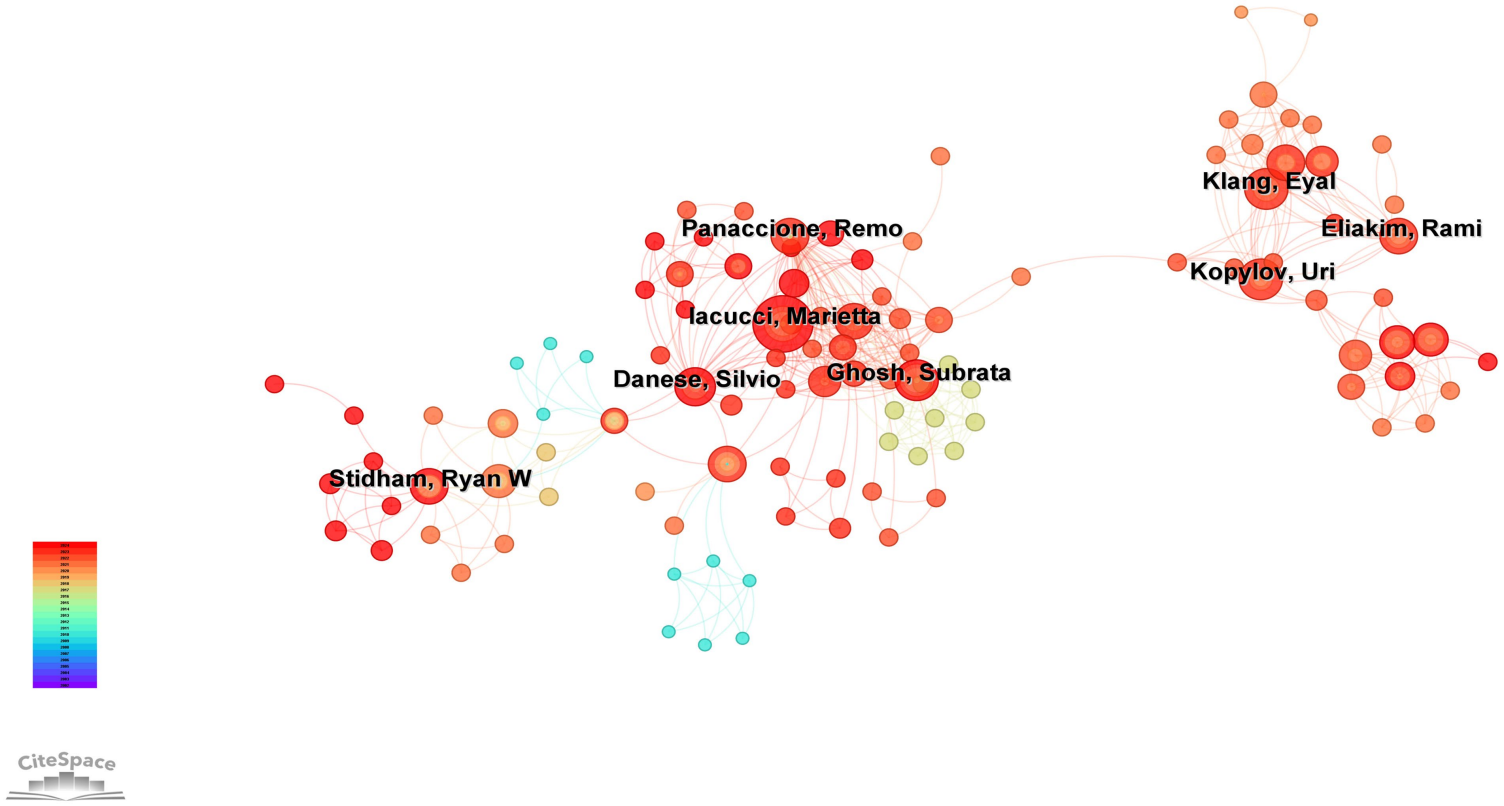
Author network visualization.

A total of 7,401 authors were included in the analysis of AI applications in IBD. Figure 3 shows the collaboration network among the authors. Based on the distribution of nodes, authors can be broadly divided into three main groups, with representative scholars including Stidham, Ryan, Iacucci, Marietta, and Kopylov, Uri. Table 1 lists the top 10 most productive authors in terms of the number of publications. Stidham, Ryan W. (21) ranks first, followed by Iacucci, Marietta (16), Waljee, Akbar (15), and Danese, Silvio (15). Regarding the *H*-index, Stidham, Ryan W. (12) ranks first, followed by Waljee, Akbar (10), Ghosh, Subrata (10), and Higgins, Peter D. R. (10). Regarding the AC/P index, Higgins, Peter D. R. (68.08) ranks first, followed by Stidham, Ryan W. (44.1) and Waljee, Akbar (43.54).

**Table 1:** The top 10 most productive authors related to the research about application of AI in inflammatory bowel disease.

Rank	Authors	Count	Percentage (%)	*H*-index	AC/P
1	Stidham, Ryan W.	21	1.85%	12	44.1
2	Iacucci, Marietta	16	1.41%	9	31.69
3	Waljee, Akbat	15	1.32%	10	57.6
4	Danese, Silvio	15	1.32%	7	17.4
5	Kopylov, Uri	13	1.14%	8	25.15
6	Ghosh, Subrata	13	1.14%	10	26.69
7	Klang, Eyal	13	1.14%	7	24.23
8	Vermeire, Severine	13	1.14%	10	43.54
9	Eliakim, R.	13	1.14%	7	25.08
10	Ben-Horin, Shomron	12	1.06%	8	26.67
11	Higgins, peter D. R.	12	1.06%	10	68.08

### 3.3 Analysis of institutions

**Figure 4:**
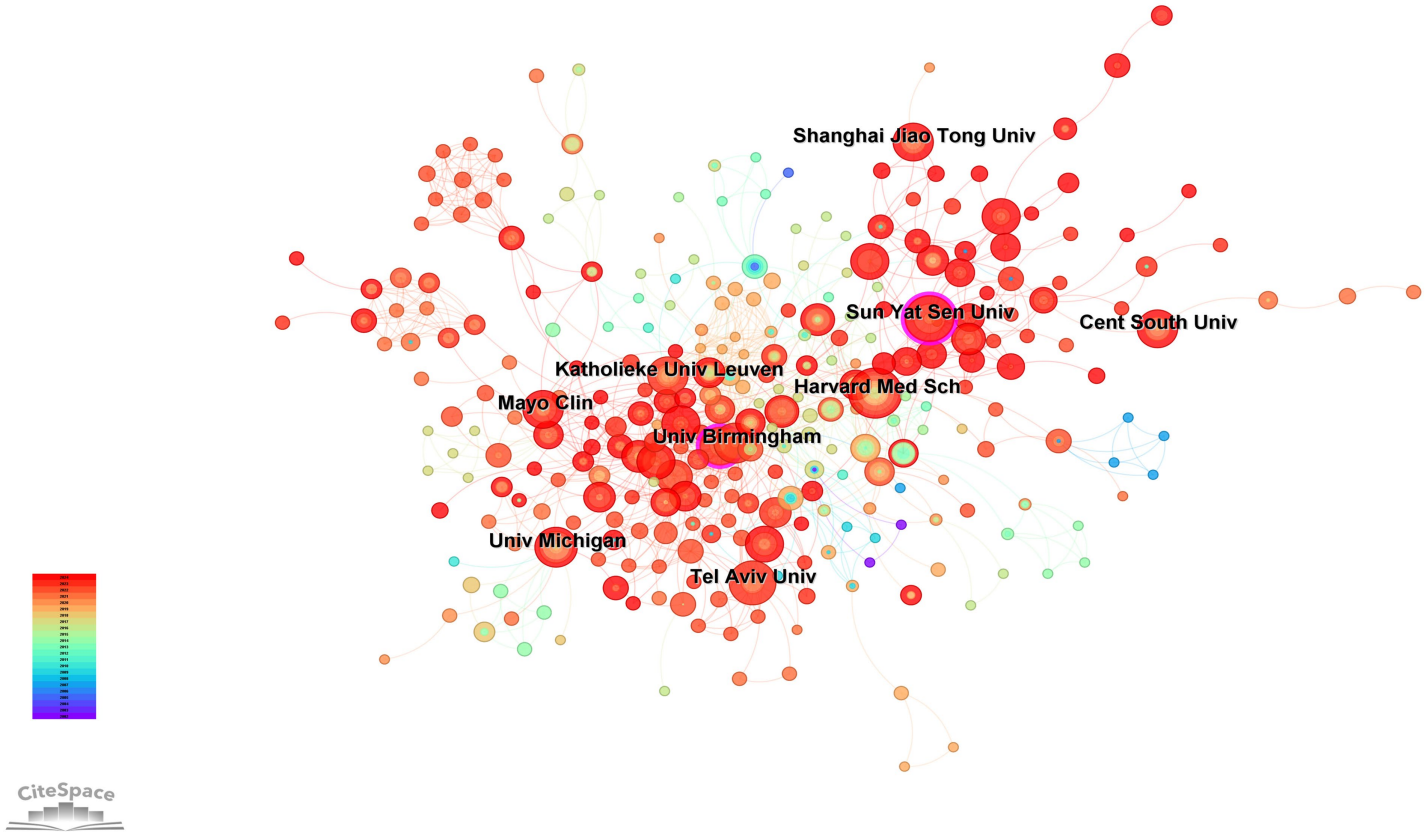
Institution network visualization.

More than 4,000 institutions have contributed to research on the AI in IBD. A total of 445 nodes were identified using CiteSpace analysis (Figure 4). Table 2 lists the 10 most productive institutions based on the number of publications. The top three institutions in terms of the number of publications and *H*-index were Harvard University (*n* = 47, *H*-index = 24), University of California System (*n* = 42, *H*-index = 18), and University of Michigan (*n* = 32, *H*-index = 17). The top three institutions in terms of AC/P were Massachusetts General Hospital (81.91), Harvard University (64.34), and the University of Michigan (48.16).

**Table 2:** The top 10 most productive institutions related to the research about application of AI in inflammatory bowel disease.

Rank	Institutions	Countries	Count	Percentage (%)	*H*-index	AC/P
1	Harvard University	USA	47	4.14%	24	64.34
2	University of California System	USA	42	3.70%	18	22.24
3	University of Michigan	USA	32	2.82%	17	48.16
4	Tel Aviv University	Israel	30	2.64%	10	18.67
5	Ku Leuven	Belgium	29	2.55%	15	33.14
6	Chaim Sheba Medical center	Israel	26	2.29%	9	16.08
7	Sun Yat-sen University	China	26	2.29%	9	11
8	US Department of Veterans Affairs	USA	25	2.20%	15	38.36
9	Massachusetts General Hospital	USA	22	1.94%	16	81.91
10	University of Birmingham	United Kingdom	22	1.94%	10	27.27

### 3.4 Analysis of countries/regions

**Figure 5:**
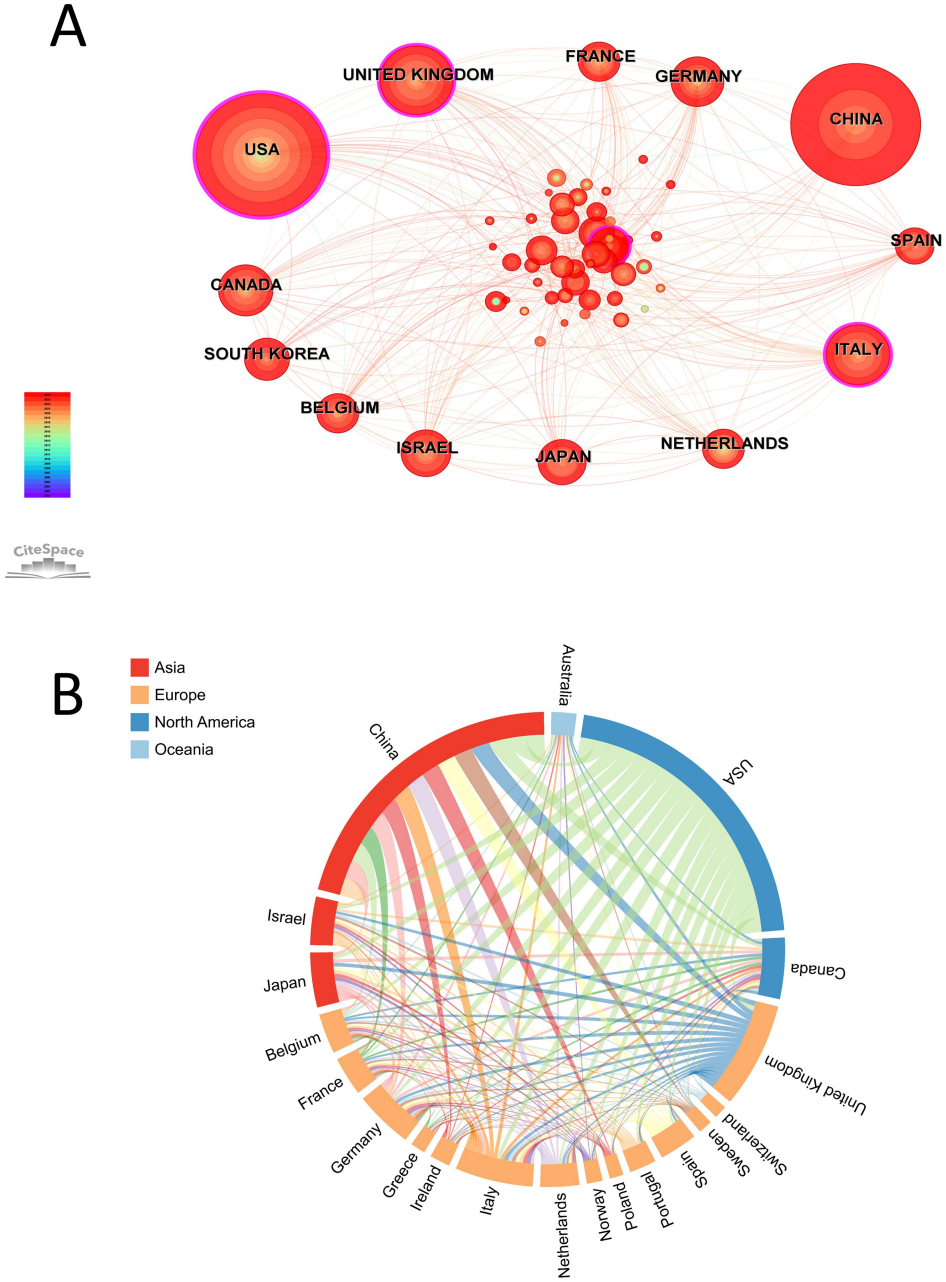
**(A)** Country/region network visualization. **(B)** Country/region cross chord charts.

A total of 65 countries/regions have published papers in related fields. Figure 5A shows the collaboration network among the countries/regions. Network analysis revealed that countries such as the USA, United Kingdom, and Italy were among the first to publish research on AI in the context of IBD. However, in recent years, the number of publications from China has grown rapidly. The top 10 most productive countries, ranked by the number of published papers, are listed in Table 3. The USA leads in terms of publication numbers, AC/P, and the *H*-index, with publications comprising 29.67% of the global total. China (327) and the United Kingdom (115) ranked second and third, respectively, although the number of publications from each country was less than 100. Figure 5B shows the collaboration among the top 20 countries, with the closest collaboration between Canada and the USA, followed by the United Kingdom and the USA, and China and the USA. Thus, the USA maintains a high level of collaboration with multiple countries globally. In contrast, collaborative relationships among other countries are relatively weak, especially across continents.

**Table 3:** The top 10 most productive countries related to the research about application of AI in inflammatory bowel disease.

Rank	Countries	Count	Percentage (%)	*H*-index	AC/P
1	USA	337	29.67%	57	33.37
2	China	327	28.79%	26	9.13
3	United Kingdom	115	10.12%	28	22.37
4	Italy	84	7.39%	21	19.99
5	Canada	66	5.81%	22	31.21
6	Germany	63	5.55%	23	25.21
7	Japan	59	5.19%	18	25.24
8	Israel	52	4.58%	15	18.17
9	South Korea	47	4.14%	11	7.7
10	Belgium	43	3.79%	18	32.53

### 3.5 Analysis of journals

A total of 458 journals published studies on the application of AI in IBD. The top 15 most productive journals are listed in Table 4. Scientific Reports published the most articles (*n* = 44), followed by Inflammatory Bowel Diseases (*n* = 41) and PLoS One (*n* = 27). Inflammatory Bowel Diseases had the highest *H*-index of 19. Data on the Impact Factors (IF) and Journal Citation Reports (JCR) quartiles were obtained from the JCR. Among the top 15 journals, Gastroenterology had the highest impact factor (IF = 25.7), and all journals were in Q1, except for Diagnostics and Frontiers in Microbiology, which were in Q2.

**Table 4:** The top 15 most productive journals related to the research about application of AI in inflammatory bowel disease.

Rank	Journal	Count	*H*-index	IF2023	JCR quartile
1	Scientific Reports	44	12	3.8001	Q1
2	Inflammatory Bowel Diseases	41	19	4.5003	Q1
3	PLoS One	27	12	2.8997	Q1
4	Diagnostics	26	7	2.9997	Q2
5	Journal of Crohn’s & Colitis	24	11	8.3001	Q1
6	Frontiers in Immunology	22	7	5.6998	Q1
7	Frontiers in Medicine	17	6	3.1000	Q1
8	Gastroenterology	15	13	25.7011	Q1
9	Journal of Clinical Medicine	15	7	2.9997	Q1
10	Therapeutic Advances in Gastroenterology	15	5	3.9003	Q1
11	World Journal of Gastroenterology	15	9	4.300	Q1
12	Frontiers in Microbiology	13	7	4.000	Q2
13	Gastrointestinal Endoscopy	12	9	6.700	Q1
14	Alimentary Pharmacology & Therapeutics	11	10	6.600	Q1
15	BMC Bioinformatics	11	8	2.900	Q1
16	Gut Microbes	11	6	12.200	Q1

### 3.6 Analysis of references

Table 5 presents the 10 most-cited articles. The article by Kieft et al. (18), published in Microbiome in 2020, had the highest number of citations (*n* = 472). This study introduced VIBRANT, which significantly improved the recovery quality of viral genomes using neural networks and a new v-score metric. This revealed specific viral populations associated with CD, providing a new perspective on microbiome-host interactions. Additionally, the Sequence Kernel Association Test method, proposed by Ionita-Laza et al. (19), combined genome-wide association studies (GWAS) and whole-exome sequencing data to enhance the effectiveness of genetic research on diseases such as CD. This method paved the way for new applications of AI in genetic data analysis. The article by Lee et al. (20) was ranked third and introduced a sequence-based gkm-SVM algorithm. This algorithm accurately predicted the impact of regulatory variants using deltaSVM scores, aiding in the identification of functional regulatory variants associated with autoimmune diseases.

**Table 5:** Most cited documents.

Rank	Title	Author	Types	Journal	Publication year	Total citations
1	VIBRANT: automated recovery, annotation and curation of microbial viruses, and evaluation of viral community function from genomic sequences	Kieft, K.	Article	Microbiome	2020	472
2	Sequence kernel association tests for the combined effect of rare and common variants	Ionita-Laza, I.	Article	American Journal of Human Genetics	2013	330
3	A method to predict the impact of regulatory variants from DNA sequence	Lee, D.	Article	Nature Genetics	2015	304
4	Gut microbiome function predicts response to anti-integrin biologic therapy in inflammatory bowel diseases	Ananthakrishnan, A. N.	Article	Cell Host & Microbe	2017	298
5	A comparative study of the gut microbiota in immune-mediated inflammatory diseases does a common dysbiosis exist?	Forbes, J. D.	Article	Microbiome	2018	284
6	Effect of a deep-learning computer-aided detection system on adenoma detection during colonoscopy (CADe-DB trial): a double-blind randomised study	Wang, P.	Article	Lancet Gastroenterology & Hepatology	2020	264
7	A meta-analysis of the utility of C-reactive protein, erythrocyte sedimentation rate, fecal calprotectin, and fecal lactoferrin to exclude inflammatory bowel disease in adults with IBS	Menees, S. B.	Article	American Journal of Gastroenterology	2015	258
8	Robust replication of genotype-phenotype associations across multiple diseases in an electronic medical record	Ritchie, M. D.	Article	American Journal of Human Genetics	2010	243
9	Human disease-drug network based on genomic expression profiles	Hu, G. H.	Article	PLoS One	2009	234
10	Reduced diversity and altered composition of the gut microbiome in individuals with myalgic encephalomyelitis/chronic fatigue syndrome	Giloteaux, L.	Article	Microbiome	2016	232

**Figure 6:**
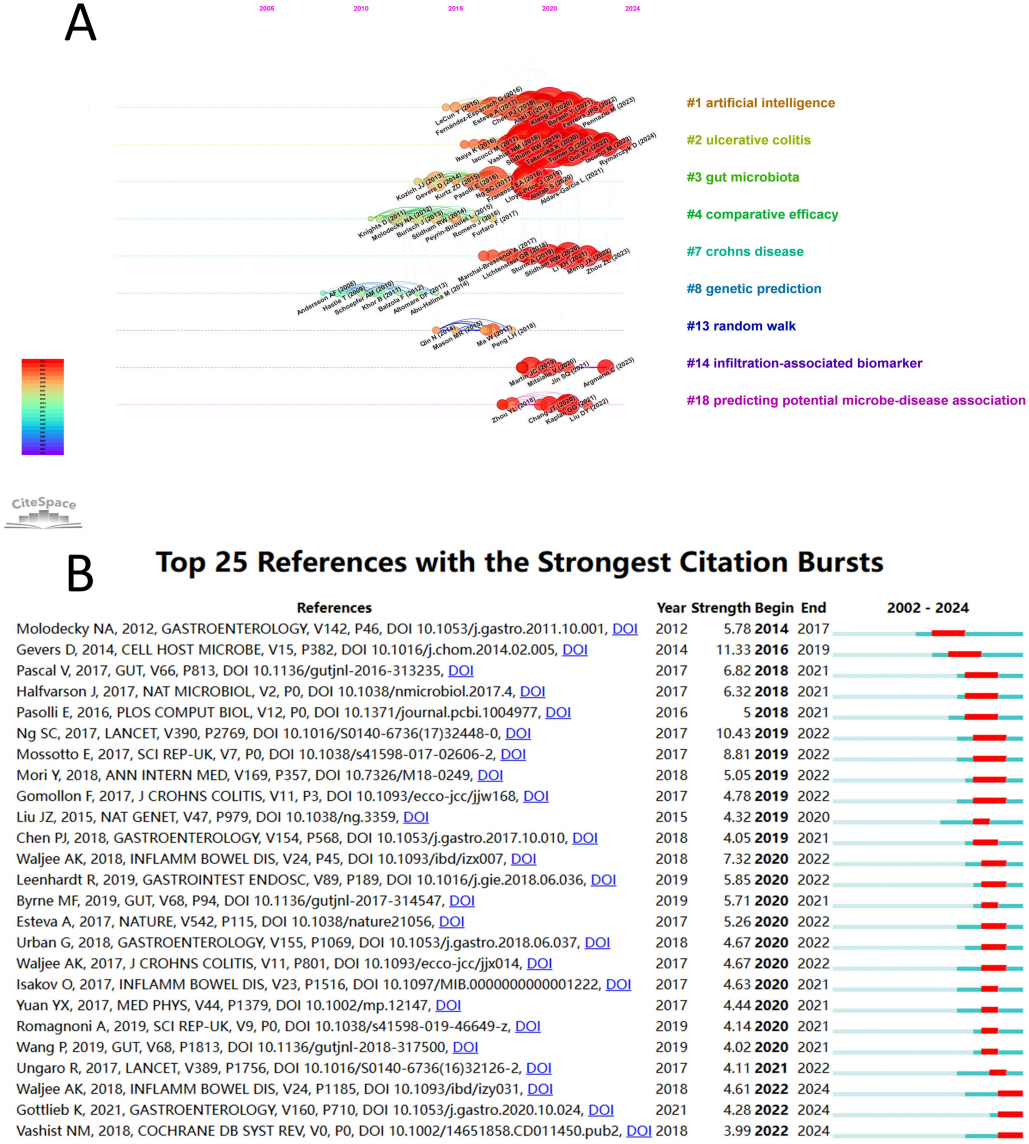
**(A)** Reference timeline visualization map. **(B)** Top 25 references with the strongest citation bursts.

A reference timeline map was constructed using CiteSpace (Figure 6A), with references clustered based on title words. The *Q* value was 0.8151 and the *S* value was 0.9269, indicating significant and highly reliable clustering results. Nine clusters were obtained: “#1 artificial intelligence,” “#2 ulcerative colitis,” “#3 gut microbiota,” “#4 comparative efficacy,” “#7 Crohn’s disease,” “#8 genetic prediction,” “#13 random walk,” “#14 infiltration-associated biomarker,” and “#18 predicting potential microbe-disease association.” References with higher citation burst intensity typically have broader scientific impact and greater influence on subsequent research. Figure 6B shows the 25 references with the strongest citation bursts. The three references with the highest burst strengths were: “The treatment-naive microbiome in new-onset Crohn’s disease” (strength = 11.3) (21), “Worldwide incidence and prevalence of inflammatory bowel disease in the 21st century: a systematic review of population-based studies” (strength = 10.43) (4), and “Classification of Pediatric Inflammatory Bowel Disease using Machine Learning” (strength = 8.81) (22).

### 3.7 Analysis of keywords

**Figure 7:**
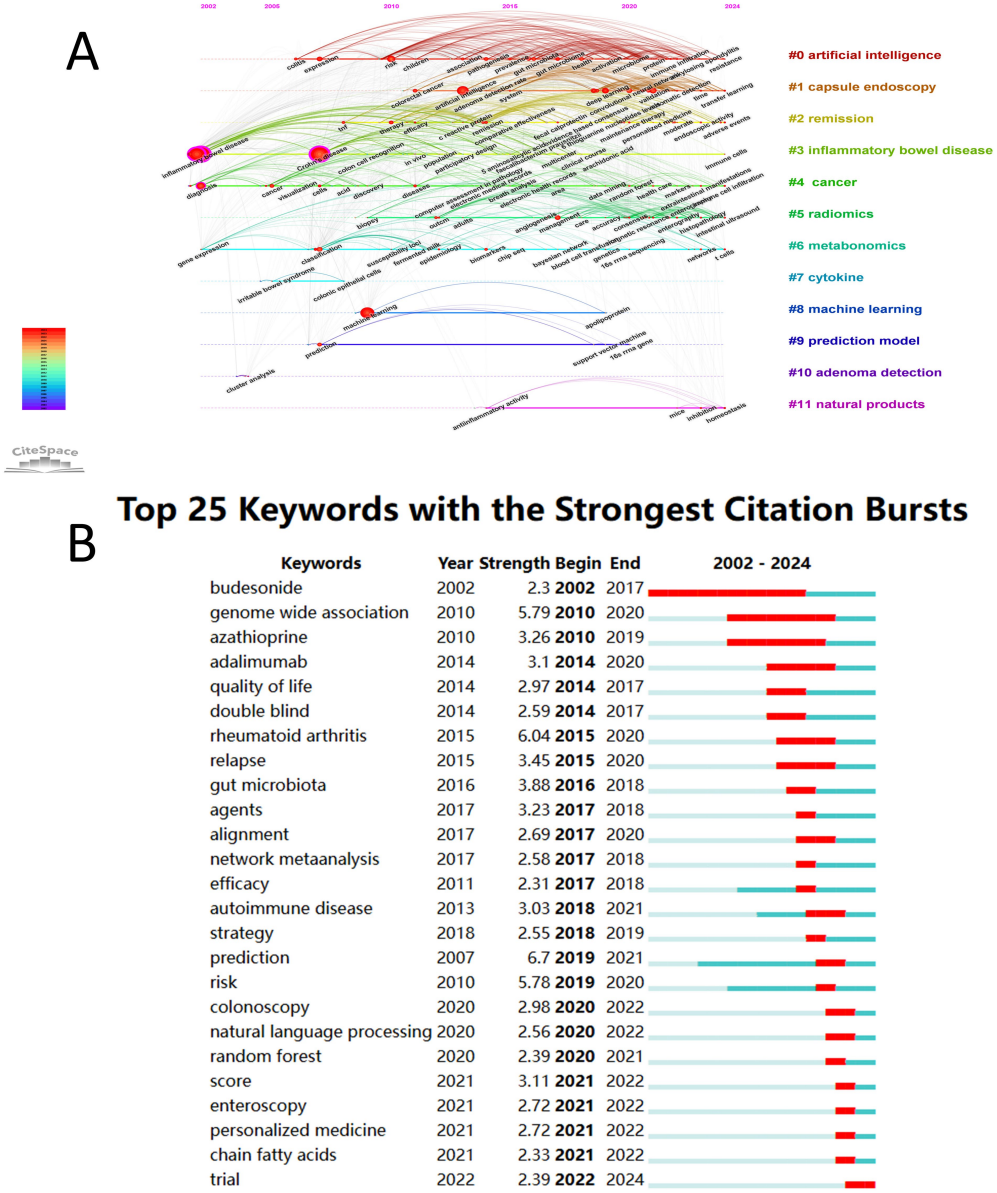
**(A)** Keyword timeline visualization map. **(B)** Top 25 keywords with the strongest citation bursts.

The 626 keywords were distributed across 12 clusters with reliable clustering results (Figure 7A) (*Q* = 0.4725, S = 0.7551). The clusters, ranked from largest to smallest, were “#0 artificial intelligence,” “#1 capsule endoscopy,” “#2 remission,” “#3 inflammatory bowel disease,” “#4 cancer,” “#5 radiomics,” “#6 metabolomics,” “#7 cytokine,” “#8 machine learning,” “#9 prediction model,” “#10 adenoma detection,” and “#11 natural products.” The timeline visualization of keywords illustrates the evolution of research development. Over time, the research hotspots have gradually shifted from IBD, cancer, metabolomics, and adenoma detection to remission, capsule endoscopy, radiomics, and natural products. Keyword burst detection was used to identify keywords with a rapid increase in frequency over a short period, thereby revealing research hotspots and emerging trends. Figure 7B shows the 25 keywords with the strongest citation bursts. The keywords that have emerged since 2020 include colonoscopy, natural language processing (NLP), random forest, score, enteroscopy, personalized medicine, short-chain fatty acids, and clinical trials.

**Figure 8:**
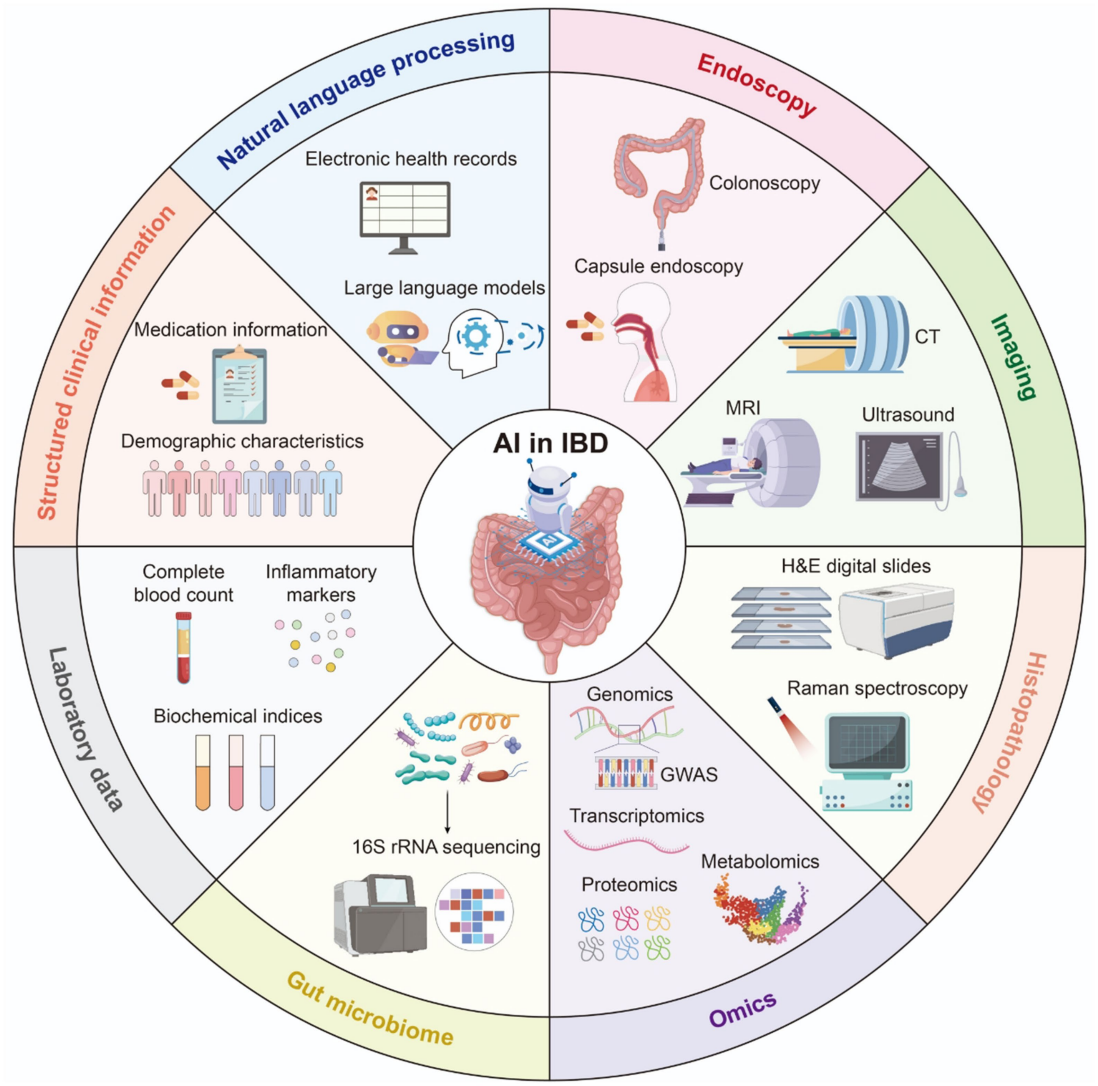
The principal investigation modalities used in AI-IBD research.

Figure 8 illustrates the eight principal investigation modalities applied in AI research on IBD, summarizing the full spectrum of data types and analytical approaches identified in our systematic analysis. These modalities include endoscopy, imaging, histopathology, omics, gut microbiome, laboratory data, structured clinical information, and NLP.

## 4 Discussion

In this study, we analyzed 1,136 publications on the application of AI in IBD retrieved from the WOSCC. The number of publications in this field showed a rapid growth trend, with 81.86% of all the papers published in the last 5 years. This rapid expansion reflects the presence of several concurrent drivers. First, the open sourcing of deep learning frameworks, such as TensorFlow in 2015 and PyTorch in 2017, has removed licensing barriers and enabled gastroenterology teams to train convolutional neural networks and transformer models on colonoscopy images, radiology scans, and electronic health record data. Second, the EU Horizon 2020 program on “AI for Health Imaging” has supported multiple multinational collaborative studies. The Notice of Special Interest NOT-CA-24-031 by NIH encourages the analysis and clinical validation of AI tools across various disease domains. Public–private initiatives such as “AI for Health” by Microsoft further lowered costs for small and mid-sized research centers by providing cloud computing resources, datasets, and engineering support. Third, strategic policy signals and data-sharing infrastructures such as the 2017 New Generation Artificial Intelligence Development Plan by China prioritized medical AI. Finally, the SECURE-IBD registry established during the COVID-19 pandemic generated large-scale clinical datasets of patients with IBD, significantly shortening the model development cycles. Given the persistence of these enabling factors, publications on AI in IBD are expected to increase rapidly.

The top 10 authors, institutions, and countries were mainly concentrated in Europe, North America, and Asia. The top 10 countries in terms of publication volume are all developed countries or major economies, suggesting a close relationship between the level of economic development and the distribution of academic output; higher economic development promotes academic output in AI applications for IBD. The outstanding performance of USA in AI applications for IBD is attributed to its early leading position in AI research, ample research funding, diverse population providing rich data resources, comprehensive policy and regulatory support, advanced medical infrastructure, and extensive international collaborations. Collectively, these factors contribute to the USA’s leading position and academic output in this field. Notably, China has also demonstrated rapid growth in academic output (Figure 5A). As of 2024, the total annual publication volume for China has reached 327 articles, approaching that of the USA. These changes reflect China’s rapid progress in this field. However, China has a relatively low *H*-index and AC/P, due to the recent rapid development of AI, which has not allowed sufficient time for publications to accumulate high citation counts. Therefore, Chinese scholars must focus on enhancing their research impact and increasing international collaboration and exchange.

Additionally, the incidence of IBD appears to have influenced the distribution of current research outputs. North America has the highest annual standardized incidence rate (ASR) of IBD, whereas Oceania has the lowest ASR. The East Asian region had the highest average annual growth rate in IBD incidence, whereas the Central European region experienced the most rapid decline in ASR (23). This trend mirrors the distribution of research output on AI in IBD, indicating a close relationship between changes in incidence rates and research output.

AI in IBD is closely associated with the collaborative development of gut microbiome studies and cancer research (Figures 6A, 7A). AI can significantly enhance the ability to identify biomarkers and therapeutic targets by integrating the gut microbiome data. Our results are consistent with the bibliometric analyses conducted by Zhang et al. (24) on IBD and gut microbiota, who indicates that countries such as the USA, China, and Italy, along with institutions such as Harvard Medical School, University of California, and University College Cork, are major contributors to research on IBD and gut microbiota. Furthermore, our results suggest that AI has played a crucial role in related studies on IBD and cancer. Bibliometric analyses conducted by Zhang et al. (25) similarly showed that countries such as China, Germany, Japan, the United Kingdom, and the USA are major contributors to research on colorectal cancer and IBD. Moreover, the increasing number of annual publications from China observed in this study are consistent with increasing research in the fields of IBD and colorectal cancer in China since 2018.

Among the top 15 journals in terms of publications, all are Q1 journals except for “Diagnostics” and “Frontiers in Microbiology,” which are ranked Q2. This suggests that AI in IBD has received widespread attention and achieved high recognition of its quality. The predominance of Q1 journals reflects the profound impact and academic value of AI in IBD. When selecting a journal for submission, researchers should prioritize highly productive and influential journals, as they provide an important platform for AI in IBD and ensure that research findings are recognized and disseminated to a broader academic community.

Based on the timeline view of references and keywords combined with burst detection analysis, this research can be broadly categorized into three distinct periods:

In the first phase (before 2012), the application of AI in IBD research was in its early stages, with fewer than 10 annual publications. Early studies relied primarily on single-modality data, such as genetic, histopathological, omics, or endoscopic data, to construct diagnostic models for IBD or IBD-related cancers or adenomas, focusing mainly on predicting the efficacy of steroid therapies. Artificial neural networks successfully distinguished IBD-related colorectal tumors from sporadic adenomas/carcinomas in the field of genetic analysis (26). Whereas, the combination of Raman spectroscopy and support vector machines achieved 98.9% accuracy in distinguishing among CD, UC, and healthy tissues in the field of histopathology (27). Decision-tree ensemble algorithms in omics research have successfully identified biomarkers (28). Additionally, endoscopic image analysis has shown great potential in clinical applications. Based on the morphological features of ulcers, a classification and regression tree (CART) model achieved 92% accuracy in distinguishing Behçet’s disease from CD (29). In terms of treatment, the CART algorithm analysis revealed that BclI and NALP1 gene polymorphisms are closely related to steroid response, emphasizing the role of genetic and demographic factors in predicting steroid efficacy in young patients with IBD (30). Moreover, with the expansion of data volume and optimization of technology, these research directions remain popular topics in the field. However, these studies did not fully utilize the synergistic effects of multimodal data. Therefore, in the future, research on AI should focus on a deeper integration of multimodal data to further enhance the accuracy and generalization ability of models, aiming to overcome the translational bottleneck from laboratory research to clinical application.

In the second phase (2012–2019), the application of AI in IBD research grew rapidly. This phase is marked by the widespread application of machine learning algorithms in areas such as drug therapy, gut microbiota, and GWAS. Predictive models for drug therapy provided strong support for personalized therapy and early intervention in clinical settings. This enabled the prediction of treatment outcomes using immunosuppressants and biologics. For example, a dynamic risk assessment system based on a random forest algorithm predicted the risk of hospitalization and steroid use in patients with IBD by integrating predictive factors such as demographic characteristics, laboratory indicators, and medication information (31). The model effectively predicted remission outcomes of thiopurine treatment in patients with IBD by analyzing laboratory indicators and age, leading to a reduction in steroid use, hospitalization, and surgery rates (32). Another algorithm based on week 6 laboratory data from vedolizumab treatment identified patients with CD who could achieve steroid-free remission by week 52 (33). The AI model provided new perspectives and methods for the early diagnosis and prognostic assessment of IBD by analyzing changes in the gut microbiota. Particularly, the machine learning model distinguished patients with CD from healthy controls based on differences in microbial communities, indicating that patients with IBD exhibited distinct dysbiosis characteristics that significantly differed from those of other immune diseases (34). The neural network algorithm vedoNet, which integrated the gut microbiome and clinical data, significantly enhanced the ability to predict clinical remission following vedolizumab treatment. These results suggested that early dynamic changes in the microbiome could serve as key markers of the IBD treatment response (35). Furthermore, GWAS revealed genetic factors associated with diseases by analyzing large-scale associations between individual genetic variations and diseases or traits. A model trained using machine learning on data from a known GWAS study of IBD-related genes successfully identified 347 new potential IBD risk genes and provided new insights into the pathogenesis of IBD (36). The gkm-SVM method, proposed by Lee et al. (20), accurately identified GWAS SNPs and their functional regulatory roles in the genomic context using deltaSVM technology and provided a powerful tool for discovering new IBD risk SNPs. However, this stage of research faced various challenges, including data heterogeneity, limited model generalizability, and difficulties in clinical translation. Interdisciplinary collaboration and big data sharing were the key drivers of the advancement of AI in IBD research.

In the third phase (after 2020), research on AI in IBD is expected to undergo rapid growth. Research hotspots during this period will primarily focus on radiomics, endoscopy, NLP, and personalized medicine. Radiomics involves the high-throughput extraction of quantitative features from medical imaging. When combined with machine learning algorithms, these features can uncover potential biological information and provide new perspectives for disease diagnosis, prognosis evaluation, and treatment decision-making. For instance, the radiomic features of magnetic resonance enterography, when integrated with clinical variables and simplified magnetic resonance activity index scores, can effectively predict the timing of surgery for CD (37). Additionally, radiomics models based on computed-tomography enterography have demonstrated clinical superiority in distinguishing intestinal fibrosis in CD, with diagnostic accuracy significantly surpassing that of traditional visual assessments by radiologists (38). Moreover, in the field of endoscopy, AI can systematically and objectively evaluate mucosal inflammation through endoscopic imaging and histological analysis, predict prognosis, and guide disease management (39). The analysis of images and videos during colonoscopy to assess UC demonstrates high accuracy, effectively evaluating the disease severity, risk of recurrence, and inflammatory activity (40–42). Furthermore, a meta-analysis indicated that AI systems exhibit high diagnostic performance in detecting mucosal healing in UC, although moderate-to-high heterogeneity exists. Thus, standardized and shared AI training helps reduce variability between systems (43). Additionally, capsule endoscopy (CE) is primarily used in CD as a crucial non-invasive diagnostic technique for examining the small intestine. Since assessing CD activity, distinguishing mucosal inflammation, and identifying complications such as strictures and fistulas is essential (44). AI can be used to identify mucosal lesions in CD, including ulcers, erosions, and strictures, by analyzing CE images (45, 46). In contrast, NLP effectively supplements the information gaps in traditional biomarkers by parsing unstructured text from electronic medical records, thereby significantly enhancing the ability to identify treatment complications (47). Breakthroughs in large language models have opened new research opportunities for NLP. For instance, ChatGPT provides accurate and comprehensive responses to real-world inquiries from patients with IBD (48). Large language models can automatically identify adverse events from clinical records and provide timely and precise data to support clinical decision-making (49). Consequently, in the future, more research based on large language models will focus on assisting with differential diagnoses, medication adjustment, efficacy monitoring, and personalized education and support. Nonetheless, issues such as linguistic diversity, data privacy and security, lack of transparency, and over-reliance in clinical applications need to be addressed. Although personalized medicine, in which treatment strategies are tailored based on individual biological characteristics, is becoming increasingly relevant for IBD management, current IBD medications, such as non-specific anti-inflammatory drugs or biologics, can only target a single aspect of the complex multifactorial biological processes. Thus, research using AI is driving the treatment of IBD from broad-spectrum anti-inflammatory drugs for phenotypically similar but biologically heterogeneous patients towards omics-based, highly specific, customized personalized medicine targeting patients with molecularly homogeneous IBD (50).

Despite the rapid expansion of AI research and applications in IBD, several unresolved challenges impede its translation from laboratory to clinical practice. These include the absence of standardized data formats, limited model interpretability and robustness, high risk of overfitting during training, and difficulty in reconciling patient privacy with data security. For instance, radiomics investigations are often confined to single-center, small-sample cohorts. Heterogeneity in imaging devices and scanning protocols obstructs cross-platform validation, and observer bias introduced by manual segmentation further undermines reproducibility. Additionally, AI-endoscopic systems suffer from biased data distributions due to variability in operator skill and image quality, which reduces algorithmic generalizability across populations. NLP models navigate through colloquial expressions and inconsistent terminology in multilingual electronic health records. Despite their promise for patient consultation and literature synthesis, large language models remain prone to hallucinations, lack rigorous fact citations, incur high computational costs, and operate within ambiguous regulatory frameworks. Multi-omics modeling is challenged by inconsistent sampling time points and divergent sequencing platforms, resulting in unstable feature integration. Moreover, most studies rely solely on single-time point retrospective data and lack longitudinal designs with multiple follow-up visits, limiting the ability of AI tools to accurately reflect disease progression and deliver reliable prognostic predictions. Consequently, clinical investigators should establish multicenter prospective databases governed by unified data collection and annotation standards to overcome these obstacles. Algorithm engineers must develop automated image-segmentation methods, cross-device adaptation techniques, and comprehensive model reliability assessment tools while leveraging federated learning or differential-privacy approaches to mitigate overfitting and safeguard patient confidentiality. Furthermore, industry stakeholders and funding bodies can facilitate this progress by supporting open cross-device benchmark datasets and financing prospective cross-platform validation studies. Regulatory agencies should implement sandbox approval processes that align with data protection regulations and require the transparent disclosure of model cards and decision logic. Integrating retrieval-augmented generation and real-time monitoring systems for large language models is essential for reducing hallucination risks. Additionally, patient and public engagement through education on data sharing and the interpretation of results is equally important. Thus, AI can be fully integrated into precision diagnostics and therapeutics for IBD through the coordinated efforts of clinicians, engineers, industry partners, policymakers, and patients.

Nevertheless, this study has some limitations. First, all the data were sourced from the WoSCC and included only English-language literature. In addition, an inherent time lag exists in database indexing, meaning that some articles published in 2024 may not have been indexed, which could result in incomplete coverage of the relevant literature. Second, although every effort was made to design a comprehensive search strategy, some relevant publications were inadvertently excluded. Finally, citation frequency is heavily dependent on publication date, therefore, newly published articles may not have accrued sufficient citations, potentially introducing bias into our analysis.

## 5 Conclusion

This study demonstrated that since the beginning of the 21st century, research on AI has undergone rapid advancement in the field of IBD and has made significant breakthroughs. The USA leads the research output in this field and exerts the most significant academic influence. Harvard University’s scholarly contributions are particularly notable among academic institutions, ranking first in terms of publication volume. During the initial phases of research, studies primarily focused on developing diagnostic models for IBD and IBD-associated cancers or adenomas using single-modality data, and on predicting the therapeutic efficacy of steroids. As research progressed, hotspots shifted towards machine learning applications in drug therapy, the gut microbiome, and GWAS. Currently, the field primarily focuses on radiomics, endoscopic technologies, NLP, and personalized medicine.

## Data Availability

The original contributions presented in the study are included in the article/Supplementary material, further inquiries can be directed to the corresponding author.
