# Supplementary material for: The global academic distribution and changes in research hotspots of artificial intelligence in inflammatory bowel disease since 2000
Source: Front Med (Lausanne). 2025 Jul 11;12:1600291. doi: 10.3389/fmed.2025.1600291 (PMC12289651; doi:10.3389/fmed.2025.1600291)
Supplement: Supplementary file 1 [file Table_1.DOCX]

**Supplementary Material 1**

TS = ("Inflammatory Bowel Disease" OR "inflammatory Bowel Diseases" OR "Crohn’s Enteritis" OR "Regional Enteritis" OR "Crohn’s Disease" OR "Crohns Disease" OR "Crohn Disease" OR "Granulomatous Enteritis" OR "Ileocolitis" OR "Granulomatous Colitis" OR "Terminal Ileitis" OR "Regional Ileitis" OR "Idiopathic Proctocolitis" OR "Ulcerative Colitis" OR "Colitis Gravis") AND TS = ("Artificial intelligence" OR "Machine learning" OR "Support vector machine" OR SVM OR CNN OR RNN OR LSTM OR ResNet OR DenseNet OR Unet OR U-net OR DNN OR "Neural network*" OR "Convolutional network*" OR "Deep learn*" OR "Semantic segmentation" OR "Ensemble Learning" OR "Classification tree" OR "regression tree" OR "probability tree" OR "nearest neighbor*" OR "random forest" OR kernel OR "k-means" OR "naive bayes" OR "Reinforcement Learning" OR "Q-learning" OR "Deep Q-Network" OR DQN OR "Policy Gradient" OR "Actor-Critic" OR "Generative Adversarial Network" OR GAN OR "Transfer Learning" OR "Bayesian Network" OR "Probabilistic Graphical Models" OR "AutoML" OR "Natural Language Processing" OR NLP OR "Transformer" OR "Graph Neural Network" OR GNN OR "Attention Mechanism" OR "Self-Supervised Learning" OR "Deep Neural Networks" OR "Recurrent Neural Networks" OR ConvNet) AND FPY = (2000-2024)
